# Healthcare-Associated Ventriculitis and Meningitis: A Retrospective Analysis

**DOI:** 10.7759/cureus.19069

**Published:** 2021-10-27

**Authors:** Elisabete Coelho, Laura Costa, José Martins, Marina Costa, José E Oliveira, António Maia-Gonçalves, Luís Lencastre

**Affiliations:** 1 Intensive Care Medicine, Hospital De Braga, Braga, PRT; 2 Intensive Care Medicine, Hospital de Braga, Braga, PRT

**Keywords:** healthcare-associated infections, intensive care, brain injury, neurosurgical procedures, central nervous system infections

## Abstract

Background and objective

Healthcare-associated ventriculitis and meningitis (HAVM) is frequent in neurocritical patients and associated with significant mortality. Surgery and intracranial devices are usually necessary and may lead to infection. Classical clinical signs and cerebral spinal fluid (CSF) analysis may be unreliable. The purpose of this study was to characterize the prevalence of HAVM, risk factors, and interventions in the neurocritical population admitted in the ICU.

Methods

This was a retrospective single-center analysis of all adult neurocritical patients admitted to an ICU during a three-year period.

Results

A total of 218 neurocritical patients were included. The prevalence of HAVM was 13% and it was found to be associated with mortality. When suspected, it was not possible to exclude HAVM in 30% of the patients. HAVM was significantly associated with surgery, surgical reintervention, and brain devices. Sustained fever was the most frequent clinical sign, and it was significantly associated with unexcluded HAVM. CSF cell count was significantly higher in HAVM, though without microbiological isolation in most of the cases.

Conclusion

Brain damage, interventions, and devices may significantly alter cerebral homeostasis. Sustained fever is very frequent and may be attributed to several conditions. CSF cell count is useful for the diagnosis of HAVM. HAVM is a clinical challenge in the management of neurocritical patients with important therapeutic and prognostic implications.

## Introduction

Healthcare-associated ventriculitis and meningitis (HAVM) is linked with a wide range of incidence in the literature, varying from 1 to 25% [1-3]. Surgery and brain devices are frequently necessary to manage neurocritical patients, but they may result in contamination and colonization [4-6]. Infections can originate directly from surgical procedures themselves, either via direct inoculation from skin flora or via adjacent dissemination of infected tissues, and occasionally from a break in the sterile technique [1,3].

The diagnosis of HAVM is based on clinical signs and cerebral spinal fluid (CSF) analysis. Classical clinical signs and symptoms, such as fever, neck stiffness, and altered mental status, are insensitive and unreliable in HAVM and manifest fully only in a minority of cases [1,7]. Hemorrhage, local inflammatory reactions, and immunosuppression may drastically alter the CSF profile [8], which may be further compromised by the use of antibiotics and steroids [2,9]. In fact, no single CSF parameter can reliably predict or exclude HAVM [8]. CSF cultures are considered the reference standard and the most important test for diagnosis of HAVM but can be negative in a broad proportion of cases [1,7]. Also, a negative CSF culture does not necessarily exclude the diagnosis [9]. Additionally, in some cases, the risk related to lumbar puncture makes CSF analysis unfeasible. Therefore, the diagnosis of HAVM remains a clinical challenge, and clinicians have to frequently rely on clinical suspicion and the absence of other infection sources to establish a diagnosis.

HAVM is associated with increased hospital length of stay, cost of care, and risk of long-term neurological impairment and death in patients, making early diagnosis and treatment imperative [1,3]. As mentioned above, the diagnosis of HAVM is a clinical challenge in neurocritical patients, and the decision regarding whether to start antibiotics is a constant concern among clinicians, and it entails balancing the risk of morbidity and mortality with complications such as toxicity [2,3,7].

The purpose of this study was to characterize the neurocritical population admitted in the ICU, identify the prevalence of HAVM, as well as describe and analyze the determinant factors in diagnosing HAVM in this population (risk factors, interventions/cerebral devices, clinical signs, and CSF analysis).

## Materials and methods

We conducted a retrospective analysis of neurocritical patients admitted to an ICU during a three-year period. All neurocritical patients who were at least 18 years of age were enrolled, including elective surgery and trauma patients. The patients admitted to the ICU with the sole purpose of organ donation were excluded.

We collected data regarding sociodemographic status, previous pathological conditions, admission diagnosis, surgery, brain devices [including intracranial pressure (ICP) monitor and external ventricular shunt (EVS)], clinical and analytical criteria related to the diagnosis of HAVM, and whether antibiotics were used. We also collected data related to severity indices [Acute Physiology and Chronic Health Evaluation (APACHE) and Simplified Acute Physiology Score (SAPS)], length of stay, and mortality.

HAVM was suspected whenever the patient had signs and/or symptoms suggestive of infection without a clear focus. The diagnostic approach included clinical and analytical evaluation, active search for other infectious sites (including microbiological samples and imaging), and CSF analysis (whenever possible).

Positive microbiological samples or bacterial DNA detection by polymerase chain reaction (PCR) confirmed the diagnosis of HAVM. Presumptive HAVM was considered whenever the diagnosis was firmly assumed by clinical staff (diagnostic approach consistent with HAVM, regardless of CSF microbiology). HAVM was not excluded whenever meningeal antibiotic dosage was used but without a firmly assumed diagnosis by clinical staff (meaning conflicting results in the diagnostic approach).

Statistical analysis was performed using JASP 0.13.1 software. A p-value of 0.005 was considered statistically significant.

## Results

Population characterization

We included a total of 218 neurocritical patients. Eighteen patients were excluded (admissions for organ donation) from the total ICU population. The median age was 55 years, with more than half of the patients between the ages of 30 and 59 years. Male patients comprised 69.7% of the sample. The most prevalent cause of admission was trauma (57.8%), followed by spontaneous brain hemorrhage (23.4%). Mortality in ICU was 14.7% and 28-day mortality was 17.0%. The main characteristics of the population are summarized in Table 1.

**Table 1:** General characteristics of the patients APACHE: Acute Physiology and Chronic Health Evaluation; HAVM: healthcare-associated ventriculitis and meningitis; ICU: intensive care unit; IQR: interquartile range; SAPS: Simplified Acute Physiology Score

Characteristic	All (n=218)	HAVM confirmed (n=4)	Presumptive HAVM (n=28)	HAVM not excluded (n=18)	HAVM excluded (n=10)	HAVM not suspected (n=162)
Age, years						
Median ± IQR	55 ± 26	44 ± 12	43 ± 23	59 ± 31	53 ± 14	55 ± 25
Distribution, n (%)						
<30 years	24 (11.0)	1 (25.0)	3 (10.7)	2 (11.1)	1 (10.0)	18 (11.1)
30-59 years	115 (52.8)	2 (50.0)	19 (67.9)	7 (38.9)	7 (70.0)	72 (44.4)
60-79 years	71 (32.6)	1 (25.0)	6 (21.4)	9 (50.0)	2 (20.0)	64 (39.5)
>80 years	8 (3.67)	0	0	0	0	8 (4.94)
Sex, n (%)						
Male	152 (69.7)	3 (75.0)	20 (71.4)	14 (77.8)	7 (70.0)	111 (68.5)
Female	66 (30.3)	1 (25.0)	8 (28.6)	4 (22.2)	3 (30.0)	51 (31.5)
Severity scores, median ± IQR						
APACHE II	19 ± 8.5	20 ± 3.0	19 ± 6.5	19 ± 8	22 ± 8	18 ± 10
SAPS III	42 ± 19.5	48 ± 5.5	42.5 ± 16	49 ± 28	46 ± 18	41 ± 20
Main diagnosis, n (%)						
Trauma	126 (57.8)	2 (50.0)	10 (35.7)	10 (55.6)	4 (40.0)	102 (63.0)
Stroke	18 (8.26)	0	1 (3.57)	5 (27.8)	1 (10.0)	19 (11.7)
Spontaneous brain hemorrhage	51 (23.4)	2 (50.0)	15 (53.6)	2 (11.1)	3 (30.0)	23 (14.2)
Brain mass/lesion	18 (8.26)	0	1 (3.57)	0	2 (20.0)	15 (9.26)
Perioperative complications/iatrogenic	5 (2.29)	0	1 (3.57)	1 (5.56)	0	3 (1.85)
Length of stay (days), median ± IQR						
ICU	8 ± 8	12 ± 8	15 ± 9	11 ± 8	13 ± 8	7 ± 7
Hospital	25 ± 37	39 ± 58	50 ± 77	38 ± 48	25 ± 35	22 ± 28
Mortality in ICU, n (%)	32 (14.7)	1 (25.0)	4 (14.3)	1 (5.56)	0	27 (16.7)
Mortality at 28 days, n (%)	37 (17.0)	1 (25.0)	5 (17.9)	3 (16.7)	1 (10.0)	28 (17.3)

HAVM prevalence

The global prevalence of HAVM was 12.8%. This diagnosis was not completely excluded in 8.26%. HAVM was suspected in 25.7% of the patients. In this subgroup, the prevalence of HAVM was 50.0%, and the diagnosis was not excluded in 32.1%.

Length of stay in the ICU (p<0.001) and in-hospital (p<0.001), as well as the 28-day status (p=0.002), were significantly associated with presumptive HAVM. There was also a statistically significant association between admission diagnosis and HAVM (p<0.001), with a trend favoring HAVM in patients with spontaneous brain hemorrhages.

Risk factors

Half of the patients (50.9%) did not have any risk factors associated with the development of HAVM (Table 2). The most frequent risk factors reported were skull base fracture and diabetes. Patients with presumptive HAVM had fewer risk factors (28.6%) than those in which HAVM was not excluded (72.2%) and even the ones with unsuspected HAVM (74.7%).

There was no risk factor significantly associated with presumptive HAVM. The risk of HAVM not being excluded was more than 25% higher with skull basis fracture (p=0.018) and more than 40% higher with dural fistula (p=0.039).

**Table 2:** Risk factors for the development of HAVM in neurocritical patients HAVM: healthcare-associated ventriculitis and meningitis

Risk factors, n (%)	All (n=218)	HAVM confirmed (n=4)	Presumptive HAVM (n=28)	HAVM not excluded (n=18)	HAVM excluded (n=10)	HAVM not suspected (n=162)
Any	107 (49.1)	1 (25.0)	8 (28.6)	13 (72.2)	6 (60.0)	121 (74.7)
Immunosuppression	6 (2.75)	0	2 (7.14)	1 (5.56)	0	3 (1.85)
Alcoholism	33 (15.1)	0	1 (3.57)	3 (16.7)	2 (20.0)	27 (16.7)
Diabetes	29 (13.3)	0	1 (3.57)	1 (5.56)	1 (10.0)	26 (16.0)
Skull base fracture	53 (24.3)	1 (25.0)	4 (14.3)	9 (50.0)	2 (20.0)	38 (23.5)
Dural fistula	17 (7.80)	0	0	4 (22.2)	2 (20.0)	11 (6.80)

Interventions/brain devices

Neurosurgery (any type) was necessary for 67.0% of the patients, and almost half of them had surgery at admission in the ICU (Table 3). Surgical reintervention was needed in 11.9% of the patients. Only 13.3% of the patients did not have any brain device. ICP monitor was more frequently utilized than EVS (81.2% and 19.3% of the patients, respectively).

**Table 3:** Devices and interventions in neurocritical patients EVS: external ventricular shunt; HAVM: healthcare-associated ventriculitis and meningitis; ICP: intracranial pressure; ICU: intensive care unit; IQR: interquartile range

Devices/interventions	All (n=218)	HAVM confirmed (n=4)	Presumptive HAVM (n=28)	HAVM not excluded (n=18)	HAVM excluded (n=10)	HAVM not suspected (n=162)
Surgery, n (%)	146 (67.0)	3 (75.0)	26 (92.3)	11 (61.1)	8 (80.0)	101 (62.3)
At admission	102 (46.8)	3 (75.0)	20 (71.4)	7 (38.9)	7 (70.0)	69 (42.6)
Between day 2 and 10 in the ICU	38 (17.4)	0	6 (21.4)	3 (16.7)	1 (10.0)	28 (17.3)
After day 10 in the ICU	5 (2.29)	0	0	1 (5.56)	0	4 (2.47)
Surgical intervention, n (%)	26 (11.9)	1 (25.0)	13 (46.4)	0	2 (20.0)	11 (6.80)
ICP monitor, n (%)	177 (81.2)	4 (100)	26 (92.3)	18 (100)	9 (90.0)	124 (76.5)
Duration of ICP monitor (days), median ± IQR	6 ± 4	8 ± 2	11 ± 5	6 ± 3	10 ± 6	7 ± 4
EVS, n (%)	42 (19.3)	2 (50.0)	18 (64.3)	4 (22.2)	5 (50.0)	15 (9.26)
Duration of EVS (days), median ± IQR	8 ± 7	8 ± 0	10 ± 3	7 ± 15	9 ± 5	2 ± 3
Manipulation of EVS, n (%)	17 (7.80)	2 (50.0)	10 (35.7)	2 (11.1)	3 (30.0)	2 (1.23)

Surgery and surgical reintervention were more frequent in patients with HAVM (92.3% and 46.4%, respectively). However, surgery was also frequent in patients in whom HAVM was excluded (80%). Both surgery (p=0.001) and surgical reintervention (p<0.001) were significantly associated with HAVM. Surgical reintervention (p=0.001) was significantly associated with suspected HAVM.

The presence of an ICP monitor was not associated with HAVM (p=0.091), but it was associated with suspected HAVM (p=0.003). The number of days with ICP monitor was significantly associated with both suspected (p<0.001) and presumptive HAVM (p<0.001). The presence of EVS was significantly associated with both suspected (p<0.001) and presumptive HAVM (p<0.001). The number of days with EVS was also significantly associated with both suspected (p<0.001) and presumptive HAVM (p=0.002). Manipulation of EVS was not associated with HAVM (p=0.110).

Clinical signs of meningitis

Less than 20% of the patients did not show any clinical signs compatible with HAVM (Table 4). Sustained fever was the most frequent symptom (72.9%), followed by sustained ICP above 20 mmHg (16.1%).

**Table 4:** Clinical signs of HAVM in neurocritical patients *Central temperature >37.5 ºC refractory to acetaminophen (1 g every six hours) and physical measures (external cooling with ice sheets) ICP: intracranial pressure; HAVM: healthcare-associated ventriculitis and meningitis

Clinical data	All (n=218)	HAVM confirmed (n=4)	Presumptive HAVM (n=28)	HAVM not excluded (n=18)	HAVM excluded (n=10)	HAVM not suspected (n=162)
Any, n (%)	176 (80.7)	4 (100)	28 (100)	18 (100)	9 (90.0)	121 (74.7)
Sustained fever*, n (%)	159 (72.9)	4 (100)	28 (100)	18 (100)	8 (80.0)	105 (64.8)
New-onset impaired mental status, n (%)	13 (5.96)	0	5 (17.9)	2 (11.1)	1 (10.0)	5 (3.09)
Seizure, n (%)	8 (3.67)	1 (25.0)	1 (3.57)	0	0	7 (4.32)
New-onset neurological deficit, n (%)	13 (5.96)	0	1 (3.57)	0	0	12 (7.41)
Sustained ICP >20 mmHg, n (%)	35 (16.1)	1 (25.0)	8 (28.6)	2 (11.1)	0	25 (15.4)
Inflammatory signs of the surgical wound, n (%)	9 (4.13)	1 (25.0)	3 (10.7)	0	3 (30.0)	3 (1.85)

Sustained fever was significantly associated with suspected (p<0.001) and presumptive HAVM (p<0.001) as well as with the inability to exclude HAVM (p=0.004).

CSF analysis

CSF samples were analyzed in 13.3% of the patients, which were collected mostly by EVS (86.9%). About 20% of the samples were collected under antibiotic pressure (Table 5).

**Table 5:** CSF analysis in neurocritical patients CSF: cerebral spinal fluid; DNA: desoxyribonucleic acid; EVS: external ventricular shunt; HAVM: healthcare-associated ventriculitis and meningitis; IQR: interquartile range; LP: lumbar puncture; PCR: polymerase chain reaction

CSF analysis	All (n=218)	HAVM confirmed (n=4)	Presumptive HAVM (n=28)	HAVM not excluded (n=18)	HAVM excluded (n=10)	HAVM not suspected (n=162)
CSF sample, n (%)	29 (13.3)	4 (100)	19 (67.9)	3 (16.7)	6 (60.0)	1 (0.62)
Under antibiotic, n (%)	6 (2.75)	1 (25.0)	4 (14.3)	0	2 (20.0)	0
Harvest method, n (%)						
EVS	26 (11.9)	2 (50.0)	17 (60.7)	3 (16.7)	5 (50.0)	1 (0.62)
LP	2 (0.92)	1 (25.0)	1 (3.57)	0	1 (10.0)	0
Hygroma puncture	1 (0.46)	1 (25.0)	1 (3.57)	0	0	0
Cells, mean number ± IQR	124 ± 465	542 ± 3473	233 ± 995	122 ± 720	18 ± 56	51
Neutrophils (%), median ± IQR	69 ± 25	43 ± 7.5	70 ± 18	76 ± 1	42 ± 42	54
Proteins (g/dL), median ± IQR	1.57 ± 1.29	1.37 ± 2.26	1.73 ± 2.48	1.02	1.35 ± 1.70	1.57
Glucose (mg/dL), median ± IQR	70 ± 16	67 ± 7.5	74 ± 19	61	73 ± 24	68
Lactate (mmol/L), median ± IQR	3.65 ± 1.10	5.85 ± 2.95	3.55 ± 2.75	3.45	3.4	4.1
Positive microbiological cultures, n (%)	3 (1.38)	3 (75.0)	2 (7.14)	0	1 (10.0)	0
Bacterial DNA detection by PCR, n (%)	1 (0.46)	1 (25.0)	1 (3.57)	0	0	0

When analyzing CSF samples, cell count was significantly higher in patients with presumptive HAVM (p=0.003), with a mean cell count of 115/mL vs. 889/mL. There was no association between CSF analysis and the inability to exclude HAVM.

The positivity rate of the microbiological cultures was 10.3%. Microbiological samples and bacterial DNA detection by PCR were positive in four patients - three with presumptive HAVM, and one in whom HAVM had been excluded.

## Discussion

The neurocritical population in this study was constituted mostly by young male individuals, with a large proportion of trauma patients. The prevalence of HAVM was about 13%, and this diagnosis was significantly associated with length of stay and mortality, consistent with other reports in the literature [1,2,10]. Patients admitted with spontaneous brain hemorrhage had a significantly higher risk of presumptive HAVM. Although trauma patients were more than half of this study population, there was not a significant association with HAVM. However, HAVM was more frequently not excluded in trauma patients either.

There was no association between any risk factors and HAVM (suspected, presumptive, or not excluded), as opposed to other reports [4-6,11], which was probably related to the low global prevalence of risk factors in this population.

Interventions and brain devices were both related to suspected and presumptive HAVM, as reported by other authors [8,11,12]. The presence of an ICP monitor was associated with suspected HAVM but not with presumptive HAVM. ICP monitors, mostly intraventricular catheters, have been described as catheters with less infectious complications [11]. In this population, all ICP monitors were intraparenchymal (bolt) catheters. Surgery was significantly associated with presumptive HAVM but not with suspected HAVM, suggesting a possible contribution of other surgical factors, such as time to- and in-surgery or surgical technique to the development of HAVM [3,12,13]. Manipulation of EVS was not associated with HAVM, as reported by other studies [11,12,14,15], which can be possibly explained by a reporting bias.

Almost all patients had some clinical manifestation that could be related to HAVM, leading to a high rate of suspicion. Sustained fever was significantly associated with suspected, presumptive, and not-excluded HAVM. Neurocritical patients may have fever for several reasons (central fever, drug fever, thrombophlebitis, chemical meningitis), and, in the absence of another clear focus of infection, it may be suggestive of HAVM [7,9]. HAVM was not excluded in about 8% of the population. However, in the subpopulation with suspected HAVM, there was more than 30% of patients in whom HAVM was not excluded. Sustained fever was the only data significantly associated with unexcluded HAVM. Skull base fracture, dural fistula, and trauma patients were more frequently associated with unexcluded HAVM, although without statistical significance. These results reflect a major concern in clinical practice: these entail very difficult differential diagnoses and there is no reliable parameter that can distinguish a sustained fever related to HAVM from that related to other causes; moreover, untreated HAVM has a significant impact on morbidity and mortality. Interestingly, unexcluded HAVM was not significantly associated with length of stay or mortality.

CSF analysis is a relevant step in the approach toward suspected HAVM but is frequently not done due to its risks (e.g., aggravating intracranial hypertension, infection, impossibility to correctly position the patient due to other trauma). Moreover, normal CSF analysis may not necessarily exclude HAVM [3,9,16]. Like clinical signs, CSF cell count, glucose, and/or protein are less reliable or diagnostically useful in patients with a recent CNS injury, surgery, or device implantation [1,9,16]. In this population, CSF analysis was done in about 13% of the patients, mostly from EVS. Importantly, about 20% of the patients were already under antibiotic therapy when CSF was analyzed. CSF cell count was the only parameter significantly associated with presumptive HAVM. Other reports state that lactate is probably the most useful biomarker for HAVM [1]. There was no correlation between the inability to exclude HAVM and CSF analysis. CSF cultures are the most important test to establish the diagnosis of HAVM [9]; however, they are negative in as many as 70% of the cases [2,3]. In this population, CSF cultures were positive in less than 10% of the presumptive HAVM, which may reflect the impact of previous antibiotic therapy or are related to chemical (aseptic) HAVM [12].

The major limitation of this study is that this was a retrospective single-center analysis based on clinical records. This is particularly relevant in terms of some of the data collected, namely the presence of dural fistula, manipulation of EVS, and clinical signs, which may be underreported in clinical records. Likewise, the classification of suspected, presumptive, excluded, and not-excluded HAVM may have been affected by the same limitation. Another limitation is the small sample size of some of the groups, which is particularly relevant in the CSF analysis.

## Conclusions

Neurocritical patients, especially patients with spontaneous brain hemorrhage, have a high risk of HAVM and it is associated with significant mortality. Surgery and brain devices are the most relevant risk factors. Clinical signs are unreliable, and sustained fever is frequently associated with unexcluded HAVM. CSF analysis should be performed whenever possible. Higher CSF cell count may be useful for diagnosing HAVM.
